# Supplementary figures and images for: High-Throughput Sequencing and Expression Analysis Suggest the Involvement of Pseudomonas putida RA-Responsive microRNAs in Growth and Development of Arabidopsis
Source: Int J Mol Sci. 2020 Jul 30;21(15):5468. doi: 10.3390/ijms21155468 (PMC7432263; doi:10.3390/ijms21155468)

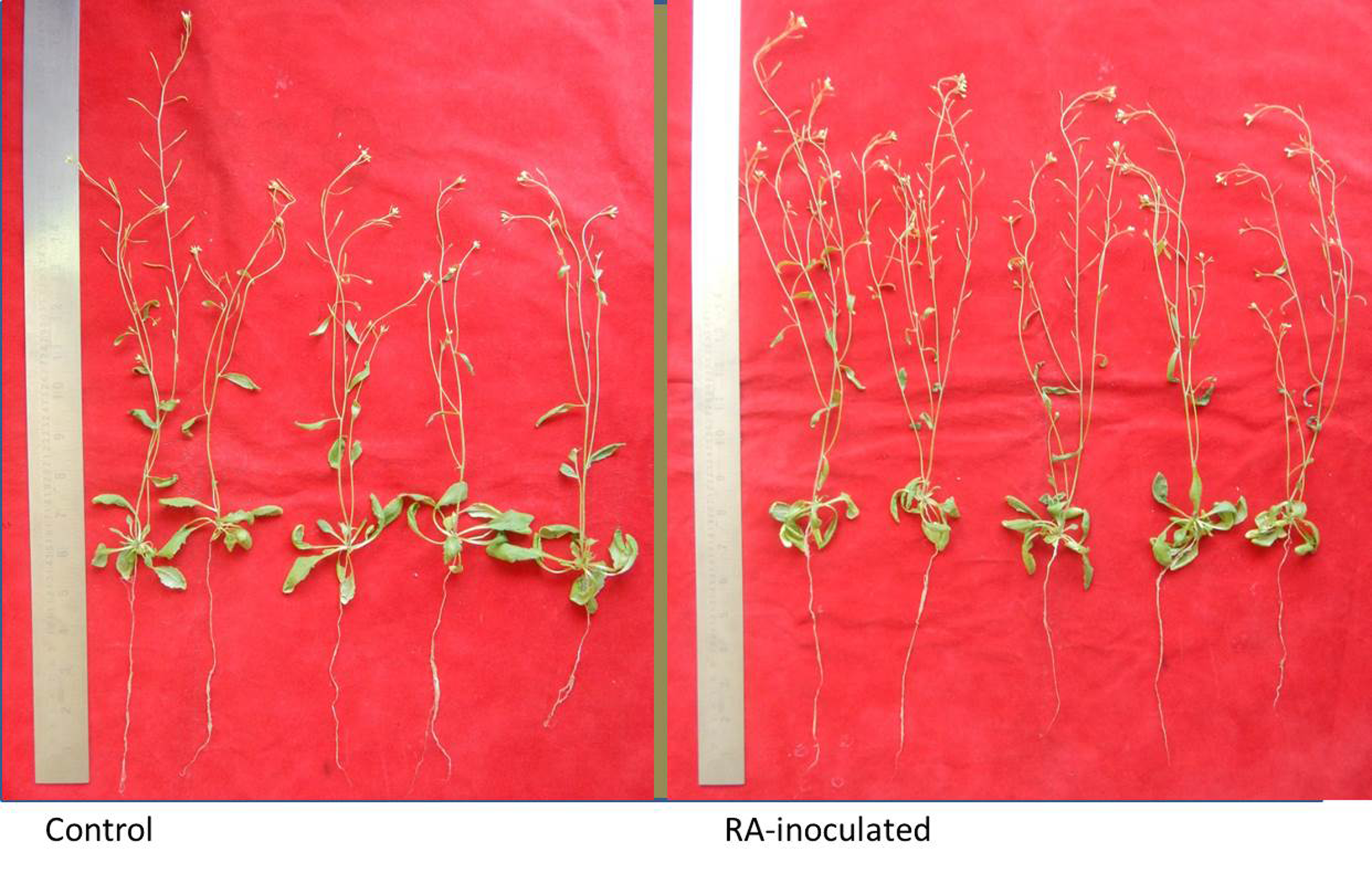

Supplement: Supplementary file 1 [file ijms-21-05468-s001.zip › Figure S1..tif]
