# Supplementary material for: High-Throughput Sequencing and Expression Analysis Suggest the Involvement of Pseudomonas putida RA-Responsive microRNAs in Growth and Development of Arabidopsis
Source: Int J Mol Sci. 2020 Jul 30;21(15):5468. doi: 10.3390/ijms21155468 (PMC7432263; doi:10.3390/ijms21155468)
Supplement: Supplementary file 1 [file ijms-21-05468-s001.zip › Table S1 miRNAs primers.doc]

**Table S1**. List of stem-loop quantitative real time PCR primers used in this study

| **miRNA ID** | **Stem-loop primer** | **Forward Primer** |
| --- | --- | --- |
| miR164a | GTCGTATCCAGTGCAGGGTCCGAGGTATTCGCACTGGATACGACCGTGCA | AATCACATGGAGAAGCAGG |
| miR164c-5p | GTCGTATCCAGTGCAGGGTCCGAGGTATTCGCACTGGATACGACCGCACG | ACATATTGGAGAAGCACGG |
| miR167b | GTCGTATCCAGTGCAGGGTCCGAGGTATTCGCACTGGATACGACTAGATT | TGATTTATGAAGCTGCCAG |
| miR319c | GTCGTATCCAGTGCAGGGTCCGAGGTATTCGCACTGGATACGACAAGGAG | TAACGATTGGACTGAAGGG |
| miR390b-3p | GTCGTATCCAGTGCAGGGTCCGAGGTATTCGCACTGGATACGACAGTTCC | AAACGACGCTATCCATCC |
| miR393a-5p | GTCGTATCCAGTGCAGGGTCCGAGGTATTCGCACTGGATACGACGATCAA | AAAGAGTCCAAAGGGATCG |
| miR396a-5p | GTCGTATCCAGTGCAGGGTCCGAGGTATTCGCACTGGATACGACCAGTTC | TACCCATTCCACAGCTTTC |
| miR398a-3p | GTCGTATCCAGTGCAGGGTCCGAGGTATTCGCACTGGATACGACAAGGGG | TCATCGTGTGTTCTCAGGT |
| miR4245 | GTCGTATCCAGTGCAGGGTCCGAGGTATTCGCACTGGATACGACACAAAG | GCACGCAATTGTCAGTATAAA |
| miR8175 | GTCGTATCCAGTGCAGGGTCCGAGGTATTCGCACTGGATACGACTGGCGC | TATTTAGATCCCCGGCAA |
| miR822-5p | GTCGTATCCAGTGCAGGGTCCGAGGTATTCGCACTGGATACGACCACATG | TAGAGTTGCGGGAAGCAT |
| miR837-3p | GTCGTATCCAGTGCAGGGTCCGAGGTATTCGCACTGGATACGACCCATCA | CCAGCTAAACGAACAAAAAAC |
| miR837-5p | GTCGTATCCAGTGCAGGGTCCGAGGTATTCGCACTGGATACGACGAAACG | CACGCAATCAGTTTCTTGTT |
| miR838 | GTCGTATCCAGTGCAGGGTCCGAGGTATTCGCACTGGATACGACTTGCAC | CCCACGCATTTTCTTCTACT |
| miR870-5p | GTCGTATCCAGTGCAGGGTCCGAGGTATTCGCACTGGATACGACTTAGAA | ACACGCAAAGAACATGAAA |
| Universal reverse primer | CCAGTGCAGGGTCCGAGGTA |  |

All primer sequences are from 5' to 3'direction.
