# Supplementary material for: High-Throughput Sequencing and Expression Analysis Suggest the Involvement of Pseudomonas putida RA-Responsive microRNAs in Growth and Development of Arabidopsis
Source: Int J Mol Sci. 2020 Jul 30;21(15):5468. doi: 10.3390/ijms21155468 (PMC7432263; doi:10.3390/ijms21155468)
Supplement: Supplementary file 1 [file ijms-21-05468-s001.zip › Table S2 Target primers.doc]

**Table S2.** List of target gene primers used in qRT-PCR analysis

| **Gene(s)** | **Accession no.^*^** | **Forward and Reverse Primer Sequences** | **Amplicon Length** | **Source** |
| --- | --- | --- | --- | --- |
| *GAMYB* | XM_004516467.2 | F-5' CGGTTGTTCTTCACCACATA 3'  R-5' GGAGTTGCAGTAGATGAATGT 3' | 120 | This study |
| *ARF18* | XM_004497453.2 | F-5' CAAAGATAACCGGCGAGAAA 3'  R-5' TCATAAGATCCGAGCGAAGA 3' | 114 | -do- |
| *ATHB-15* | XM_004486719.2 | F-5' ATCTCGCATCGGAAACTATG 3'  R-5' GTGAAACTCCGTCCAACTTA 3' | 101 | -do- |
| *BBP* | XM_004502512.1 | F-5' AGGCAGTGCATCTATAAACA 3'  R-5' CAGTGTAAGTTGCTGCATTAG 3' | 89 | -do- |
| *Laccase* | XM_004490517.2 | F-5' GTTTGTTTGGAGGCAGAGA 3'  R-5' CGACTGACAAAGGAAGCAA 3' | 104 | -do- |
| *ERF7* | XM_004513832.2 | F-5' TGAAGCCGGAGATCCATTT 3'  R-5' TGATGTTGGTCTCCGATGAT 3' | 88 | -do- |
| *PK* | XM_004502959.2 | F-5' CACCAACACCAAGCTATGTA 3'  R-5' GAAGTGCATGAGATGATGAGA 3' | 106 | -do- |
| *MAPKKK* | XM_004504680.2 | F-5' CCTGTGTCTCTTGTTGTTGA 3'  R-5' CTGAAAGAACCCGAATCAGT 3' | 110 | -do- |
| *GAPDH* | AJ010224 | F- 5' CCAAGGTCAAGATCGGAATCA 3'  R-5' CAAAGCCACTCTAGCAACCAAA 3' | 65 | Garg et al. 2010 |

*GenBankacession/Reference no.

| BAT1 | ATGTTAATGGCGACACGTA  GACAGAGTACATGAGTAAAGGA |  |
| --- | --- | --- |
| CUC1 | AATAACTCTGCTGCTCATACC  TCCCAAATCCAGAAACTGAC |  |
| DREB19 | TGAAGACGCTATTGAGCTTG  GCTGTAACCTTCCTCTGATTT |  |
| GIF2 | CCAATGGGTATGACCAACAA  ATCTGCTCCATCTTGCTTTC |  |
| GPAT4 | CGTTTGCCCGAAGAAATGA  TGCCTCCAAGCAACAGATA |  |
| GRF1 | TGGTTCTCTTTCTAACAGCAG  AGGAGCAGTAGCAGAAGTAT |  |
| LBD16 | ACGGTCATGTAAACCCTAAC  CGGTTTGATGATGAGTCTCT |  |
| NRAMP1 | TCTGTGGAATTCTTGGGTTC  AATGTCCTGTCTTGGAAGTG |  |
| NRT1 | GGGTGTGGTTAATCTGGTTT  CTTGTCCTCCTCAAAGTCTTC |  |
| SDH5 | TCCACTTGAAGATGAGATGC  CCTTTGCTCCACGTAAGAT |  |
| SZF1 | TAACGCAGCTAGAGACTACA  TATCCGAACCCTCTCATTCA |  |
| TCP4 | TTCGGAAGGATTCAGAGACTA  TAGGAGGAGCGAACAGAAA |  |
| TIR1 | TGCGATAGCTTGAGGAAAC  AACTCACGGAACAAGAAGAC |  |
| TONSOKU | AGTGTGATGATCTCGAAGTTG  GCTCTTTCACGATATGGTTCT |  |
| TUB2 | TACCAGCAATACCAAGATGC  CCTCCTGTTGATATTCACCTTC |  |
