# Supplementary material for: High-Throughput Sequencing and Expression Analysis Suggest the Involvement of Pseudomonas putida RA-Responsive microRNAs in Growth and Development of Arabidopsis
Source: Int J Mol Sci. 2020 Jul 30;21(15):5468. doi: 10.3390/ijms21155468 (PMC7432263; doi:10.3390/ijms21155468)
Supplement: Supplementary file 1 [file ijms-21-05468-s001.zip › Table S3 Morphological parameters.doc]

**Table S3**: Effect of RA-inoculation on morphological parameters of Arabidopsis

| **Treatments** | **Root length (cm)** | **Shoot length (cm)** | **No. of lateral branches per plant** | **Fresh weight (mg)** | **Dry weight (mg)** |
| --- | --- | --- | --- | --- | --- |
| **Control** | 15.86 ± 0.81 | 28.43 ± 1.84 | 3.43 ± 0.30 | 1035.71 ± 0.10 | 97.27 ± 0.01 |
| **RA-inoculated** | 18.39 ± 0.98 (p<0.01) | 35.49 ± 0.87 (p<0.001) | 4.71 ± 0.27 (p<0.01) | 1571.43 ± 0.08 (p<0.001) | 171.53 ± 0.01 (p<0.001) |

Data shown the means ± SE (n = 7 plants). Student’s t-test used to determine the significant differences in between the control and RA-inoculated plants.
